# Enzymatic characterization of a glycoside hydrolase family 5 subfamily 7 (GH5_7) mannanase from *Arabidopsis thaliana*

**DOI:** 10.1007/s00425-013-2005-y

**Published:** 2013-12-11

**Authors:** Yang Wang, Francisco Vilaplana, Harry Brumer, Henrik Aspeborg

**Affiliations:** 1 Division of Glycoscience, School of Biotechnology, KTH Royal Institute of Technology, AlbaNova University Centre, 106 91 Stockholm, Sweden; 2 Division of Industrial Biotechnology, School of Biotechnology, KTH Royal Institute of Technology, AlbaNova University Centre, 106 91 Stockholm, Sweden; 3 Wallenberg Wood Science Centre, Royal Institute of Technology (KTH), 100 44 Stockholm, Sweden; 4 Michael Smith Laboratories and Department of Chemistry, University of British Columbia, 2185 East Mall, Vancouver, V6T 1Z4 Canada

**Keywords:** GH5_7, β-Mannanase, Glycoside hydrolase, Mannan, Plant cell wall, Carbohydrates

## Abstract

Each plant genome contains a repertoire of β-mannanase genes belonging to glycoside hydrolase family 5 subfamily 7 (GH5_7), putatively involved in the degradation and modification of various plant mannan polysaccharides, but very few have been characterized at the gene product level. The current study presents recombinant production and in vitro characterization of AtMan5-1 as a first step towards the exploration of the catalytic capacity of *Arabidopsis thaliana* β-mannanase. The target enzyme was expressed in both *E. coli* (AtMan5-1e) and *P. pastoris* (AtMan5-1p). The main difference between the two forms was a higher observed thermal stability for AtMan5-1p, presumably due to glycosylation of that particular variant. AtMan5-1 displayed optimal activity at pH 5 and 35 °C and hydrolyzed polymeric carob galactomannan, konjac glucomannan, and spruce galactoglucomannan as well as oligomeric mannopentaose and mannohexaose. However, the galactose-rich and highly branched guar gum was not as efficiently degraded. AtMan5-1 activity was enhanced by Co^2+^ and inhibited by Mn^2+^. The catalytic efficiency values for carob galactomannan were 426.8 and 368.1 min^−1^ mg^−1^ mL for AtMan5-1e and AtMan5-1p, respectively. Product analysis of AtMan5-1p suggested that at least five substrate-binding sites were required for manno-oligosaccharide hydrolysis, and that the enzyme also can act as a transglycosylase.

## Introduction

Plants can create diverse cell wall composites with different properties by varying the arrangement of the four main cell wall components cellulose, hemicelluloses, lignin and pectins (Gibson 2012). Hemicelluloses, sometimes referred to as cross-linking or matrix polysaccharides, are a class of structurally heterogeneous polymers closely interacting with cellulose and lignin. Xyloglucans, xylans, mannans and mixed-linkage β-glucans are generally included in hemicelluloses category (Scheller and Ulvskov 2010). β-1,4-Mannans show a widespread distribution in plant tissues and cell wall types, and have been an important plant carbohydrate since the green algae moved out of the water and began the colonization of land. In certain cell walls, i.e., the secondary cell wall of gymnosperms and the Type III primary cell wall of ferns, mannan-type polysaccharides are the main hemicelluloses (Rodriguez-Gacio Mdel et al. 2012; Silva et al. 2011). Based on the backbone monomer composition and the presence of side chains, the mannans can be divided into four groups: mannans, glucomannans, galactomannans and galactoglucomannans. Mannans and glucomannans are linear polymers with either a backbone composed exclusively of β-1,4-linked mannosyl residues or a main chain with a varying distribution of glucose and mannose units joined together with β-1,4 glycosidic bonds. Decorating these two types of backbones with β-1,6-linked galactose side chains results in the branched galactomannans and galactoglucomannans. A further layer of complexity is the occurrence of acetylations of mannans, a modification that may mask the actual mannan polysaccharide distribution in planta (Marcus et al. 2010). Mannans are structural components of the cell wall, but can also function as energy-storage compounds in seeds, bulbs and tubers (Schroder et al. 2009). In the model plant *Arabidopsis thaliana*, mannan-type polysaccharides are detected in low levels in most tissues, but appear to be more abundant in flowers, siliques and inflorescence stems. Secondary cell walls of xylem elements, xylem parenchyma and interfascicular fibers contain higher amounts of mannans (Handford et al. 2003; Liepman et al. 2007). A detailed mannan labeling pattern in the stem was recently described showing similar features of mannan detection between Arabidopsis and poplar (Kim and Daniel 2012).

The principal enzymes involved in the degradation and/or modification of mannan-based polysaccharides are *endo*-β-mannanases (E.C. 3.2.1.78). These enzymes are responsible for catalyzing hydrolysis of the β-1,4-linked backbone within different mannans. In the CAZy (carbohydrate-active enzymes) database (Cantarel et al. 2009), enzymes with this activity are classified into three different glycoside hydrolase families: GH5, GH26 and GH113. *Endo*-β-mannanases in these GH families share a (β/α)_8_ barrel fold structure and a retaining mechanism with conserved catalytic residues (Glu: acid/base; Glu: nucleophile). All plant mannanases belong to family GH5, specifically subfamily 7 (GH5_7) (Aspeborg et al. 2012). Mannanase genes have been identified in all published plant genomes including moss (*Physcomitrella patens*), cucumber (*Cucumis sativis*), poplar (*Populus trichocarpa*), rice (*Oryza sativa*) and Arabidopsis (*Arabidopsis thaliana*). In the genome of the model plant *Arabidopsis thaliana*, the *endo*-β-mannanases family consists of eight members, but one is likely a pseudo gene (Yuan et al. 2007). The GH5_7 enzymes encoded by those genes have signal peptides and an active site with the expected conserved amino acids (Aspeborg et al. 2012; Yuan et al. 2007). Notably, plant mannanases have a sole GH5 catalytic module without any attached carbohydrate-binding module (CBM).

Plant mannanase enzymes have in general been assumed to perform hydrolysis in growth and developmental processes. Several mannanases have been associated with seed germination, whereas others have a role in flower development and fruit ripening (Bewley et al. 2000; Brummell et al. 2004; Bourgault and Bewley 2002; Filichkin et al. 2004). Recently, mannanase involvement in wounding and xylem differentiation was suggested (Yan et al. 2012; Zhao et al. 2013). Arabidopsis mannanase transcripts have been detected in all examined tissues. Interestingly, three of them (*AtMan5*–*5*, *AtMan5*–*6*, and *AtMan5*–*7*) are expressed in germinating seeds and affect the germination time (*t*_50_) (Iglesias-Fernandez et al. 2011a, b). The *AtMan5*–*1* transcript (also known as *AtMAN1* or *At1g02310*) has been detected in three different tissues (inflorescence, stem, and root), but not in geminating seeds (Yuan et al. 2007).

Only a few plant mannanases have been enzymatically characterized. LeMAN4a involved in tomato fruit development has been reported to possess both hydrolytic and transglycosylation activities (Schroder et al. 2006; Bourgault et al. 2005); and the soybean protein GmMAN1 perform hydrolysis during soybean seedling establishment (Lin et al. 2011). Product analysis of a purified mannanase extracted from barley seedling, also indicated both hydrolytic and transglycosylation reactions (Hrmova et al. 2006). As yet, the catalytic properties of Arabidopsis GH5_7 mannanases are unexplored. In an effort to increase the number of characterized plant GH5_7 enzymes, and evaluate their biotechnological potential, a detailed investigation of the properties of a recombinantly expressed *Arabidopsis* mannanase (AtMan5-1) is presented in the current study.

## Materials and methods

### Bioinformatic analysis

The protein coded by the gene with locus At1g02310 was designated AtMan5-1 in accordance with the nomenclature for glycoside hydrolases, but also to reflect the historical acronym AtMAN1 (Yuan et al. 2007; Henrissat et al. 1998). Protein sequences similar to AtMan5-1 were downloaded from the phytozome v8.0 database (http://www.phytozome.org) (Goodstein et al. 2012). Sequences were trimmed to include only the catalytic module and truncated sequences were removed. The existing Phytozome protein names were abbreviated to a format similar to the Arabidopsis locus nomenclature. Protein alignments were performed using muscle (Edgar 2004). A maximum likelihood (ML) tree was created using PhyML (Guindon and Gascuel 2003). Branch support was evaluated using bootstrap analysis with 100 replicates. N-Glycosylation sites were predicted using the NetNGlyc 1.0 server (http://www.cbs.dtu.dk/services/NetNGlyc/) and ELM (http://elm.eu.org/) (Blom et al. 2004; Dinkel et al. 2012).

### Gene cloning

Plasmid DNA containing the AtMan5-1 full-length cDNA was obtained from RIKEN (Seki et al. 1998, 2002). To express the target gene in *E. coli* (AtMan5-1e), the open reading frame of AtMan5-1 (Gene locus, At1g02310) without signal peptide was amplified by PCR using Phusion polymerase (Finnzymes) and the forward primer 5′-CACCATGGTAAAGACAGGCT-3′ and the reverse primer 5′-TTCTGCACTATGTGTGACCA-3′. The forward primer contained a CACC overhang needed for the TOPO^®^ cloning procedure. The PCR products were cloned into the pENTR/SD/D-TOPO entry vector (Invitrogen). The cloning reaction mixture was transformed into chemically competent *E. coli* TOP10 cells. The extracted constructs were recombined with the pET-DEST42 destination vector (providing a C-terminal His_6_-tag) (Invitrogen) by using the LR Clonase mix II (Invitrogen). To express the target gene in *P. pastoris* (AtMan5-1p), the catalytic module without the signal peptide were PCR amplified using the forward primer 5′-ATTATCGCGGCCGCCGTA AAGACAGGCTTTG-3′ and the reverse primer 5′-GGCGGCTCTAGATGTTCTGC ACTATGTGTGAC-3′. The constructs were combined with the pPICZα-C vector (including an α-factor secretion signal for extracellular expression of the desired protein, and a C-terminal His_6_-tag) by ligation. To confirm the presence of correct gene, DNA sequencing was performed by a commercial service (Eurofins MWG Operon).

### Protein expression and purification

To obtain expressed AtMan5-1e, the *E. coli* Rosetta (DE3) transformants were grown in Terrific Broth with 100 μg/mL ampicillin and 30 μg/mL chloramphenicol at 37 °C. The culture was induced by adding 0.5 mM isopropyl-1-thio-β-d-galactopyranoside (IPTG) at OD_600_ of 0.5–0.8 and grown at 16 °C overnight. Biomasses were harvested by centrifugation (4,750 rpm, 4 °C and 15 min). The cell pellets were resuspended in phosphate buffer (50 mM, pH 7.4) with NaCl (0.5 M) and lysed by performing French press. Crude extracts were separated from cell debris by centrifugation (20,000 rpm, 4 °C and 30 min) and then used for protein purification.

To obtain secreted AtMan5-1p, the *P. pastoris* SMD1168H transformants were grown in BMGY medium at 30 °C to an OD_600_ of 2–6. The cells were harvested by centrifugation (1,500*g*, 4 °C and 5 min) and resuspended in BMMY medium to OD_600_ of 0.5. Cultures were grown at 25 °C up to 72 h and feed with methanol to a final concentration of 1 % every 24 h. Culture supernatants were harvested by centrifugation (4,750*g*, 4 °C and 30 min) and then used for protein purification.

AtMan5-1e was purified via its C-terminal His_6_-tag by immobilized metal affinity chromatography (IMAC) using an ÄKTA purifier system (Pharmacia; Uppsala, Sweden) with a BioRad Profinity IMAC Ni-Charged Resin (10 mL; BioRad Laboratories; Hercules, USA). Then, the protein was purified further by size-exclusion chromatography (SEC) using a HiPrep 26/60 Sephacryl S-200 column (GE Healthcare) in 20 mM tris–HCl (pH 8.1), which was followed by ion exchange chromatography using HiTrap Q XL column (GE Healthcare). The eluted protein was washed and concentrated by 10 kDa cutoff Amicon Ultra centrifugal filters (Millipore) using sodium citrate buffer (50 mM, pH 5). AtMan5-1p was purified via its C-terminal His_6_-tag by IMAC as described above, and followed by buffer exchanging to sodium citrate (50 mM, pH 5.5).

To evaluate the purity and size of enzyme preparations, sodium dodecyl sulfate polyacrylamide gel electrophoresis (SDS-PAGE) was performed by using 10 % precast polyacrylamide gels (BIO-RAD, USA) and 1× Tris–Glycine buffer. Deglycosylation of AtMan5-1p was performed using the Glycoprofile™ II, Enzymatic In-solution N-deglycosylation kit (Sigma) following the manufacturer’s protocol. For identification of the generated recombinant enzymes, proteins bands of interest were excised, trypsin digested, and peptide samples were analyzed by mass spectrometry (MS) (Hale et al. 2004). MASCOT was used to process the data.

### Enzyme activity assay

Enzyme activity was determined on the basis of 3,5-dinitrosalicylic acid (DNS) reducing sugar assay (Miller 1959). Total volume of all assay reactions was 500 μL containing 3.2–7.8 ng/μL AtMan5-1e or 3.1–13.1 ng/μL AtMan5-1p. Blanks were prepared in the same way as test samples but using the 50 mM sodium citrate buffer instead of enzyme. Reducing sugars were quantified at 540 nm by a Cary 50 UV–visible spectrophotometer (Varian). To determine substrate specificity, reactions were performed by using the following polysaccharides as substrates: carob galactomannan (galactose:mannose ratio 1:4, Megazyme), konjac glucomannan (glucose:mannose ratio 1:1.5, Megazyme), guar gum (galactose:mannose ratio 1:2, Sigma) and spruce galactoglucomannan (mannose:glucose:galactose ratio 3.5–4.5:1:0.5–1.1) (Willfor et al. 2008). The released reducing sugars were measured after incubating the enzyme with these soluble mannose-based polysaccharides (3 mg/mL) in 50 mM sodium citrate buffer (AtMan5-1e: pH 5.0, AtMan5-1p: pH 5.5) at 30 °C for 30 min. The pH and temperature profiles, and the effects of metal ions were estimated by assaying mannanase activity using carob galactomannan (3 mg/mL) as substrate in 50 mM sodium citrate buffer for 30 min. The optimal pH was measured in a range of pH 3.0–7.0. The optimal temperature of enzyme activity was determined at the following temperatures: 5–45 °C with 5 °C interval. To monitor the thermal stability, the enzymes were incubated at various temperatures (15, 25, 35 and 45 °C). After incubation for 0, 30, 60, 120 and 240 min, enzyme aliquots were taken out to assay their residual activities. The influence of various metal ions on mannanase hydrolytic activity was studied by incubating the enzymes with 5 mM metal ion chloride salts (Ca ^2+^, Co ^2+^, Fe^3+^, Li ^2+^, Mg^2+^, Mn^2+^ and Ni ^2+^) in 50 mM sodium citrate buffer (AtMan5-1e: pH 5.0, AtMan5-1p: pH 5.5) containing 3 mg/mL carob galactomannan at 35 °C for 30 min. The control reaction was performed under the same conditions as above but without addition of any metal ion. To determine apparent kinetic parameters, enzymatic activity was measured in 50 mM sodium citrate buffer (AtMan5-1e: pH 5.0, AtMan5-1p: pH 5.5) containing carob galactomannan (1.5–6 mg/mL) at 35 °C (Miller 1959).

### Product analysis

To confirm the hydrolytic capability of AtMan5-1, samples were prepared using different substrates including 2 mg/mL suspensions of carob galactomannan, konjac glucomannan, guar gum, spruce galactoglucomannan, arabinoxylan, hydroxyethylcellulose, xyloglucan and 1 mM of mannotriose (M3), mannotetraose (M4), mannopentaose (M5), 6^3^,6^4^-α-d-galactosyl-mannopentaose (G_2_M5), mannohexaose (M6). 0.26 μM AtMan5-1p enzyme was added into the polysaccharide reactions, whereas the oligosaccharide reactions contained 0.13 μM enzyme. The mixtures were incubated at room temperature for 7 days. Oligosaccharide analysis was carried out by high-performance anion-exchange chromatography with pulsed amperometric detection (HPAEC-PAD) using an ICS-3000 system (Dionex, Sunnyvale, CA). 10 μL of enzyme incubations were injected in a PA200 column with an isocratic flow of 0.5 mL/min of 45 mM NaOH at 30 °C during 15 min. Peak assignation was performed by comparison of the retention time with a series of manno-oligosaccharides [mannose, mannobiose (M2), M3, M4, M5, M6] from Megazyme (Ireland).

To detect possible transglycosylation reactions, 0.22 μM of purified AtMan5-1p was mixed with 5 mM M5/M6 or 5 mM M5/M6 and 1 M sodium chloride, and these reactions were incubated at 35 °C for 24 h. The final products were subjected to matrix-assisted laser desorption/ionization time-of-flight mass spectrometry (MALDI-ToF-MS) for molar mass analysis. The reaction products and the matrix solution [10 g/L 2,5-dihydroxybenzoic acid (DHB) in 50 % v/v acetone] were pre-mixed in a ratio 1:1 and spotted directly on a standard steel MALDI plate prior to analysis. The mass spectra were collected using a LaserToF LT3 Plus instrument (SAI, Manchester, UK) in the reflectron mode with positive ionization.

## Results

### Bioinformatic analysis

**Fig. 1:**
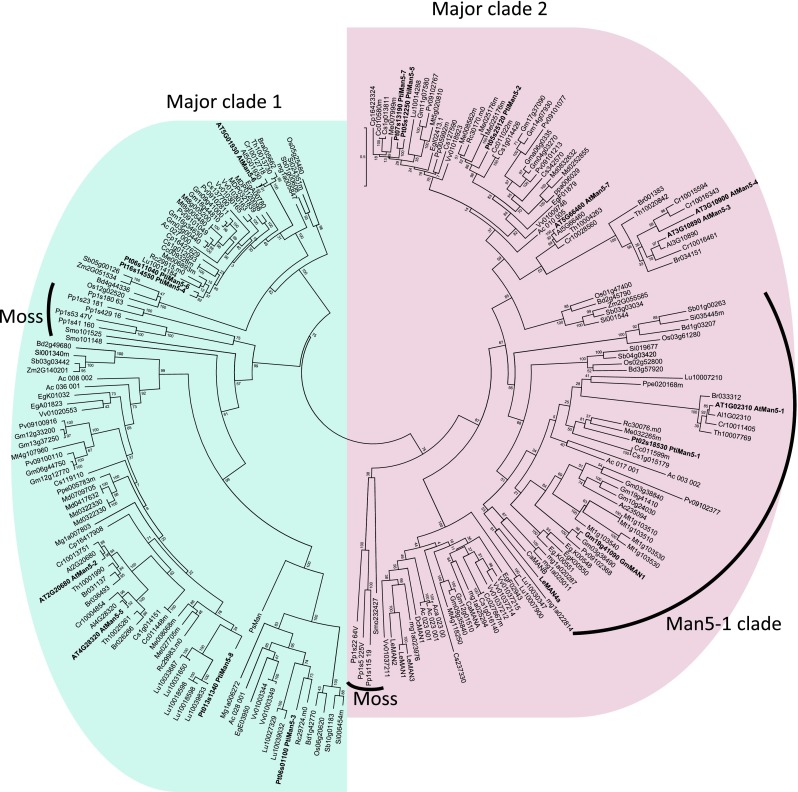
Phylogenetic analysis of plant GH5_7 protein sequences using PhyML. Only the catalytic module was used for the analysis. All poplar and Arabidopsis proteins plus GmMAN1 and LeMAN4a are marked in *bold* text. Clades containing moss and spike moss sequences are highlighted as well as the clade containing AtMan5-1, GmMAN1 and LeMAN4a. The two major clades are shaded in *blue* and *pink*. Phytozome protein names were abbreviated to resemble Arabidopsis locus names, e.g., orange1.1g014151m.csi.18105486 would be shortened Cs1g014151. To the Phytozome dataset, these sequences were added: *Coffea arabica* (CaManA = CAC08208, CaManB = CAC08442), *Picea sitchensis* (PsMan5A = ADE76368) *Solanum lycopersicum* (LeMAN1 = AF01744, LeMAN2 = AF184238, LeMAN3 = AF290893, LeMAN4a = AY046588)

Previous phylogenetic studies have shown that plant GH5_7 proteins form their own clade within subfamily GH5_7 (Aspeborg et al. 2012). To make use of the wealth of sequenced plant genomes, including sequences not deposited in Genbank, and get a deeper insight into plant mannanase evolution, plant GH5_7 mannanase protein sequences were downloaded from the Phytozome database and a few additional mannanase sequences from coffee (*Coffea arabica*; GenBank accession CAC08208, CAC08442), sitka spruce (*Picea sitchensis*; GenBank accession ADE76368) and tomato (*Solanum lycopersicum*; GenBank accession AY046588, AF01744, AAG14352, AF184238) were added to the dataset. The Arabidopsis mannanases were redesignated so that the GH family number is included in their names (Henrissat et al. 1998). A phylogenetic analysis of the catalytic segments of the proteins revealed two major clades, and in each clade there was a group of moss (*Physcomitrella patens*) and spikemoss (*Selaginella moellendorffii*) proteins (Fig. 1). Arabidopsis GH5_7 sequences are located in both clades. AtMan5-1 belongs to the same subclade (denoted Man5-1 clade in Fig. 1) as the characterized enzymes LeMAN4a and GmMAN1. However, AtMan5-1 together with a few other Brassicaceae proteins constitute an outgroup of that particular subclade.

**Fig. 2:**
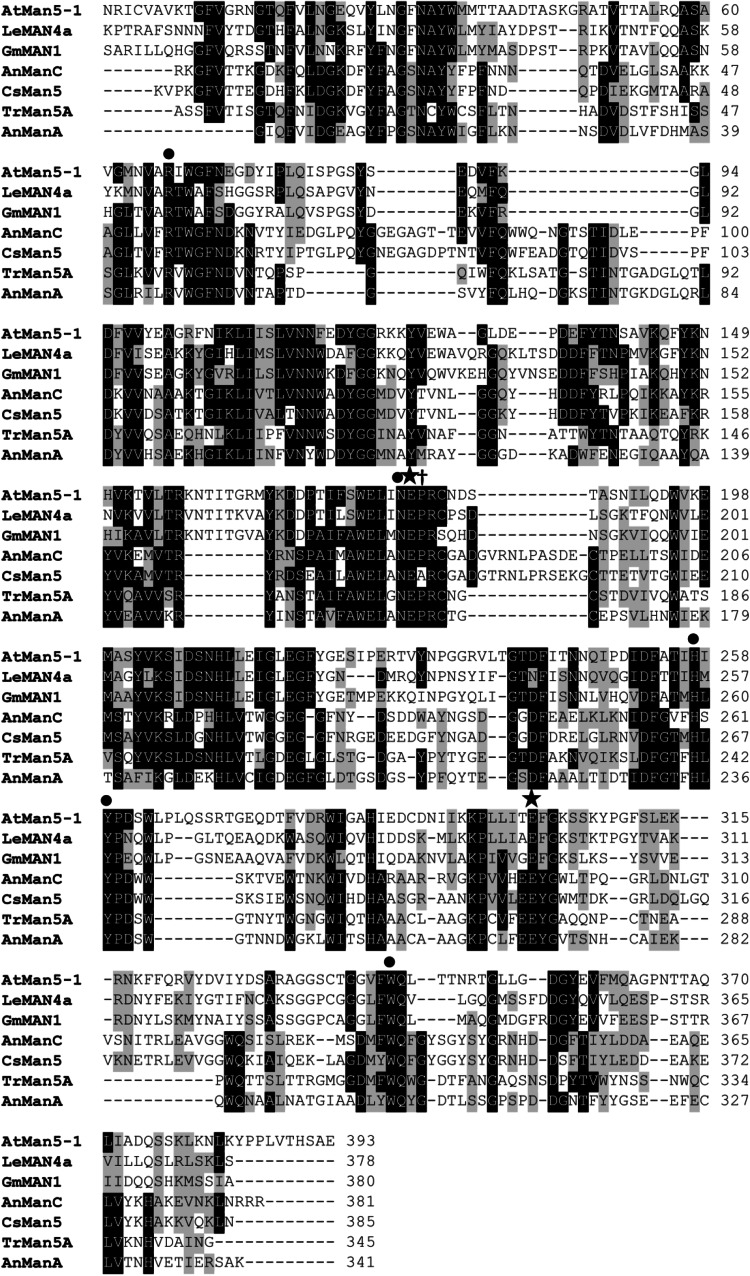
Sequence alignment of AtMan5-1 together with the plant mannanases GmMAN1 and LeMAN4a. In addition, fungal sequences have been included from *Chrysonilia sitophila*, *Aspergillus niger* and *Trichoderma reesei*. Catalytic glutamates are marked with a *star* and other highly conserved GH5 amino acids are highlighted with a *black filled circle*. The arginine located at the +2 subsite is marked with a *cross*

The AtMan5-1 protein consists of 411 amino acids. There are two predicted glycosylation sites according to the NetNGlyc 1.0 server, one at the N-terminus and the second one at the C-terminus, whereas the ELM program suggests a site at the C-terminus, although a different sequon as compared with the NetNGlyc prediction. By sequence alignment analysis the catalytic glutamates can easily be identified as (E198) and (E319), as well as other well conserved residues within the GH5 family (Fig. 2) (Jenkins et al. 1995; Henrissat et al. 1996). Most amino acids suggested to be involved in substrate binding of LeMAN4A seem to be maintained in AtMan5-1 (Dilokpimol et al. 2011; Bourgault et al. 2005). For instance, an arginine (R200) at the +2 subsite important for transglycosylation capability is conserved in AtMan5-1 (Rosengren et al. 2012). However, differences can also be observed. The LeMAN4a tryptophan (W135) and the glutamine (Q282) located at the +1 subsite are substituted with a phenylalanine and a serine, respectively, whereas a serine (S369) and a phenylalanine (F370) positioned around subsite −3 in the LeMAN4a structure have been replaced with two leucines in AtMan5-1 (Fig. 2). Notably, all of the Brassicaceae GH5_7 proteins in the five-membered outgroup clade including AtMan5-1 are lacking an aromatic amino acid at the position corresponding to (F370) in the protein alignment.

### Recombinant protein expression and purification

**Fig. 3:**
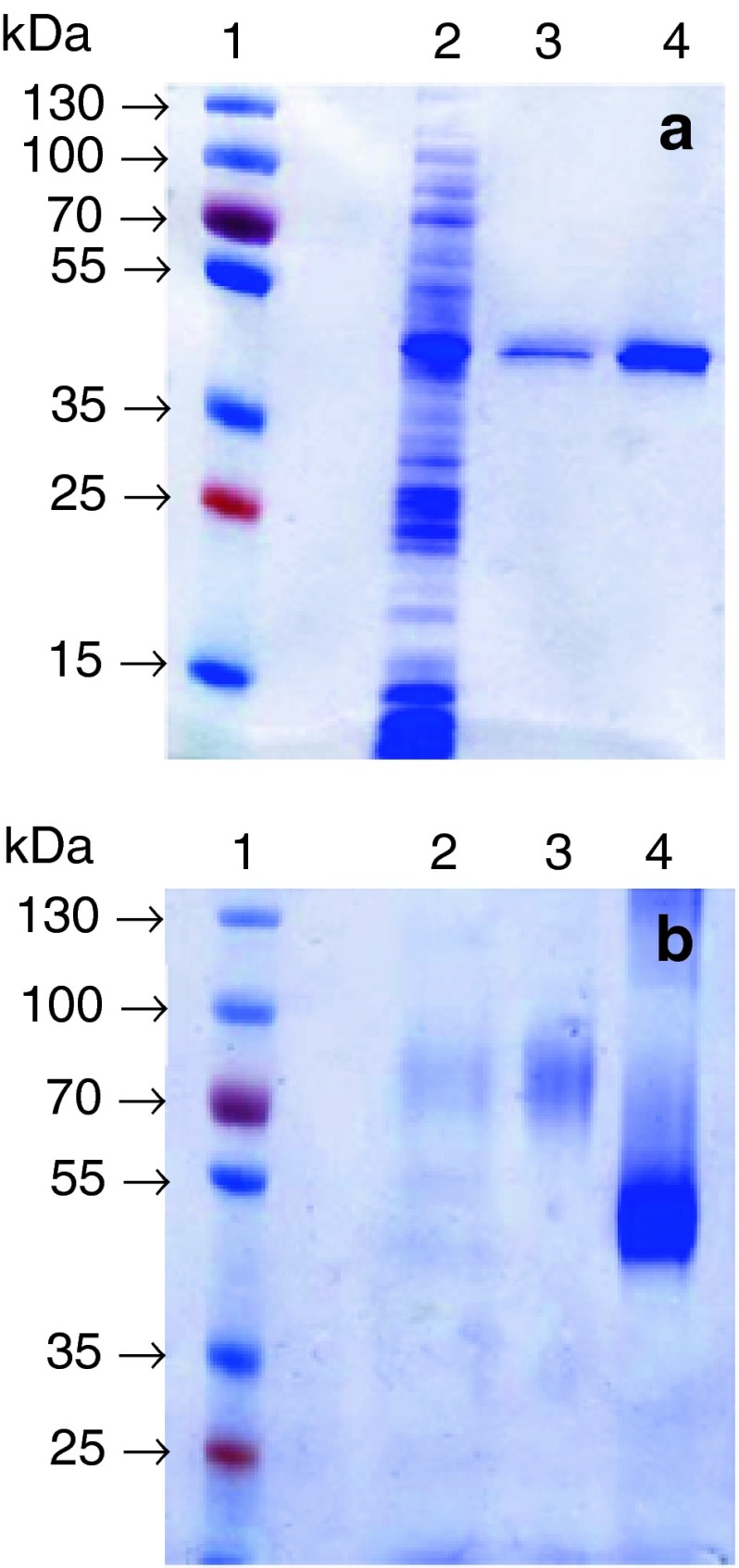
SDS-PAGE analysis of AtMan5-1. **a** AtMAN5-1e: *lane 2*, crude extract before purification; *lane 3* and *lane 4*, 0.7 and 2.2 μg purified recombinant protein, respectively. **b** AtMan5-1p: *lane 2* crude extract before purification; *lane 3* 9.6 μg purified recombinant protein before deglycosylation with PNGase F; *lane 4* 9.6 μg purified recombinant protein after deglycosylation with PNGase F

AtMan5-1 with a C-terminal His_6_ tag was successfully expressed in both *E. coli* Rosetta (DE3) cells (denoted AtMan5-1e) and *P. pastoris* SMD1168H cells (denoted AtMan5-1p). The purification yield was higher for AtMan5-1p (12 mg/L) than for AtMan5-1e (0.7 mg/L). The recombinant AtMan5-1e was purified by IMAC followed by gel filtration and ion exchange. The recombinant AtMan5-1p was purified in a single step using IMAC. According to SDS-PAGE, AtMan5-1e migrated as a single distinct band of approximately 48 kDa (Fig. 3a), which was consistent with the calculated molecular weight (48.7 kDa). The band of the purified AtMan5-1p was not as clear, but the size was estimated to be approximately 85 kDa before deglycosylation and 50 kDa (calculated: 48.2 kDa) after removing the glycan moiety (Fig. 3b). The non-deglycosylated AtMan5-1p was used in the following activity characterizations. The identities of the purified proteins were confirmed with protein in-gel digestion by trypsin followed mass spectrometry (MS) analysis. Four unique peptides (RQASAVGMNVARI; RTGEQDTFVDRW; RVYDVIYDSARA; KGLDFVVYEAGRF) perfectly matched the AtMan5-1 sequence.

### Recombinant enzyme characterization

A range of substituted and non-substituted mannans including carob galactomannan, konjac glucomannan, guar gum and spruce galactoglucomannan were used to screen the substrate specificity of AtMan5-1. Recombinant AtMan5-1 expressed from both hosts hydrolyzed all tested mannan polysaccharides, but the activity on guar gum was considerably lower (Table 1). AtMan5-1e exhibited the highest activity toward carob galactomannan, whereas AtMan5-1p was most active on konjac glucomannan. However, for AtMan5-1p the determined specific activities for carob galactomannan, konjac glucomannan, and spruce galactoglucomannan were approximately at an equal level.

**Table 1:** Substrate specificity of AtMan5-1

Substrate (3 mg/mL)	Substrate specificity (U/mg)	
	AtMan5-1e	AtMan5-1p
Carob galactomannan	5.94 ± 0.07	2.22 ± 0.13
Konjac glucomannan	3.81 ± 0.02	2.61 ± 0.24
Guar gum	0.94 ± 0.01	0.96 ± 0.01
Galactoglucomannan	2.96 ± 0.04	2.20 ± 0.20

**Fig. 4:**
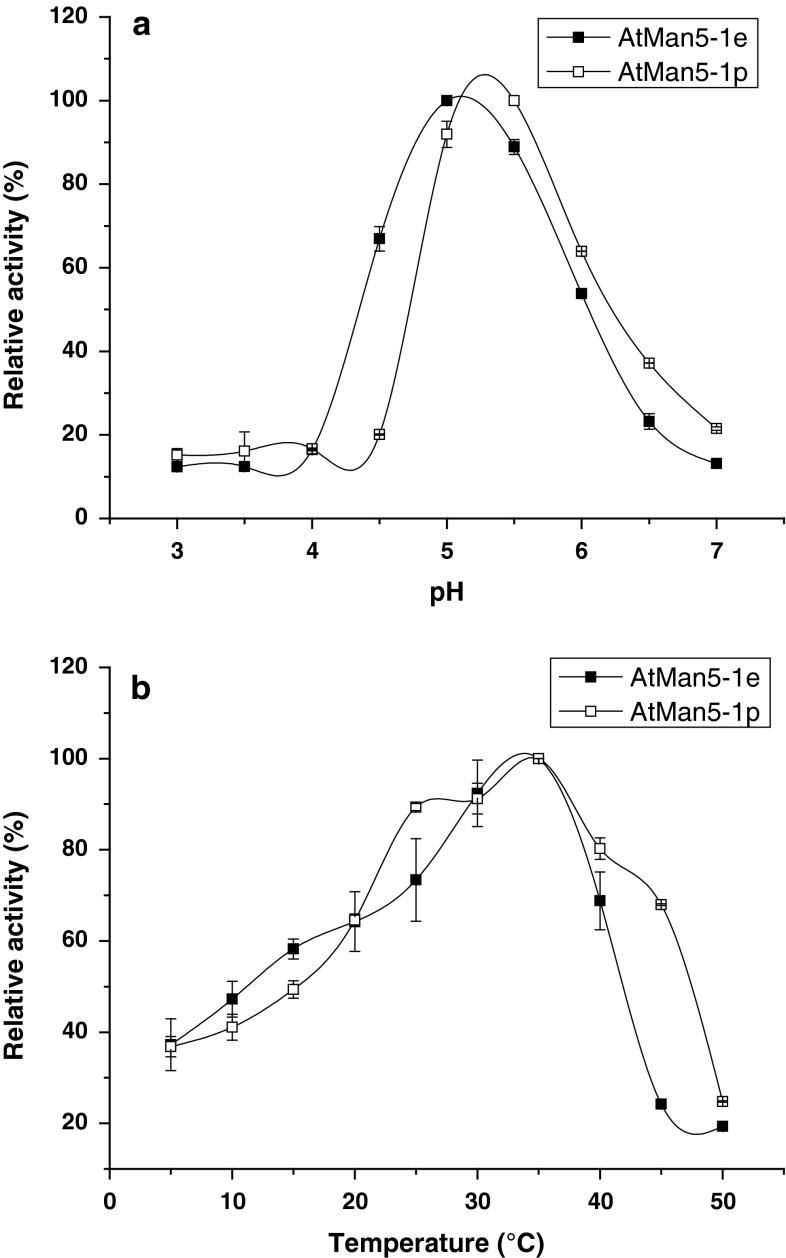
**a** pH profile of AtMan5-1e and AtMan5-1p. AtMan5-1 activity at optimal pH was set as 100 % activity. **b** Temperature profile of AtMan5-1e and AtMan5-1p. The AtMan5-1 activity at optimal temperature was set as 100 % activity

**Fig. 5:**
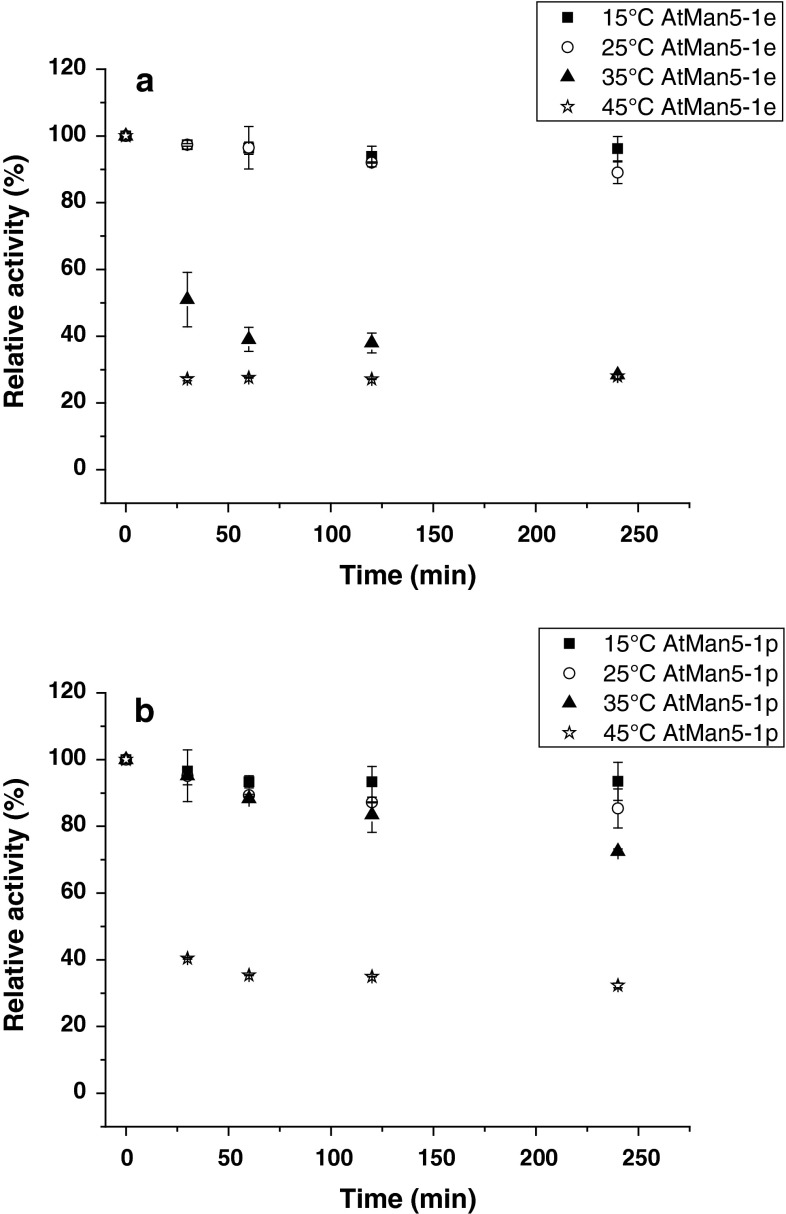
The thermal stability of AtMan5-1. **a** AtMan5-1e and **b** AtMan5-1p were incubated at 15 °C (*squares*), 25 °C (*circles*), 35 °C (*triangles*), and 45 °C (*stars*), respectively. The AtMan5-1 activity without incubation was set as 100 % activity

The obtained pH profiles showed typical bell-shaped curves, and the apparent pH optima for the recombinant enzymes using carob galactomannan as substrate were pH 5.0 and pH 5.5 for AtMan5-1e and AtMan5-1p, respectively (Fig. 4a). The temperature effect on hydrolytic activity was assayed at different temperatures (0–45 °C). AtMan5-1e and AtMan5-1p showed the highest level of catalytic activity at 35 °C (Fig. 4b). The thermal stability of AtMan5-1 was determined by measuring the residual enzyme activity after incubation at various temperature and time intervals. AtMan5-1p was slightly more thermostable than AtMan5-1e (Fig. 5). At 35 °C AtMan5-1p retained most its activity, whereas AtMan5-1e showed a 50 % activity loss already at 30 min at the same temperature.

**Fig. 6:**
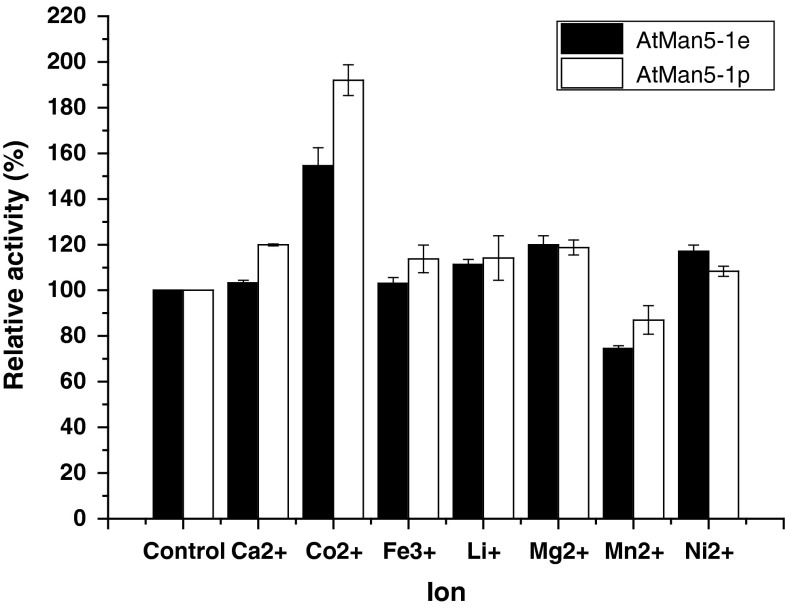
Ion influence on the activity of AtMan5-1e and AtMan5-1p. The AtMan5-1 activity without adding ion was set as the control with 100 % activity

The influence of metal ions on AtMan5-1 activity was measured by incubating the enzyme with various metal ions. AtMan5-1e and AtMan5-1p showed a similar response in this experiment. The ions Li^+^, Mg^2+^ and Ni^2+^ slightly increased the activity of both AtMan5-1e and AtMan5-1p, whereas a moderately enhanced activity could only be observed for AtMan5-1p incubated with Ca^2+^ and Fe^3+^ (Fig. 6). The most pronounced increase of activity was detected in the presence of Co^2+^, and the only metal ion displaying an inhibitory effect on mannanase activity in this investigation was Mn^2+^.

The kinetic parameters of AtMan5-1 were determined by using carob galactomannan as substrate under optimal conditions. The Michaelis constant *K*_m_ was expressed in mg/mL since carob galactomannan is a polydisperse substrate. The determined *V*_max_, *K*_m_ and *k*_cat_ */K*_m_ of values of AtMan5-1 are presented in Table 2. The catalytic efficiency (*k*_cat_ */K*_m_) was slightly higher for the *E. coli* produced AtMan5-1 (AtMan5-1e *k*_cat_ */K*_m_ = 426.8 min^−1^ mg^−1^ mL, AtMan5-1p *k*_cat_ */K*_m_ = 368.1 min^−1^ mg^−1^ mL).

**Table 2:** Biochemical characterization and kinetic comparison of AtMan5-1

Enzyme	Molecular mass (kDa)		Apparent optimal pH and temperature		*V*_max_ (U/mg)	*K*_m_ (mg/mL)	*k*_cat_ */K*_m_ [mL/(min mg)]
	Theoretical	Experimental	pH	Temperature (°C)			
AtMan5-1e	48.7	≈48	5.0	35	65.4 ± 2.4	7.4 ± 0.3	426.8 ± 1.9
AtMan5-1p	48.2	≈85/≈50	5.5	35	60.6 ± 1.8	9.5 ± 0.4	368.1 ± 3.5

### Product analysis

**Fig. 7:**
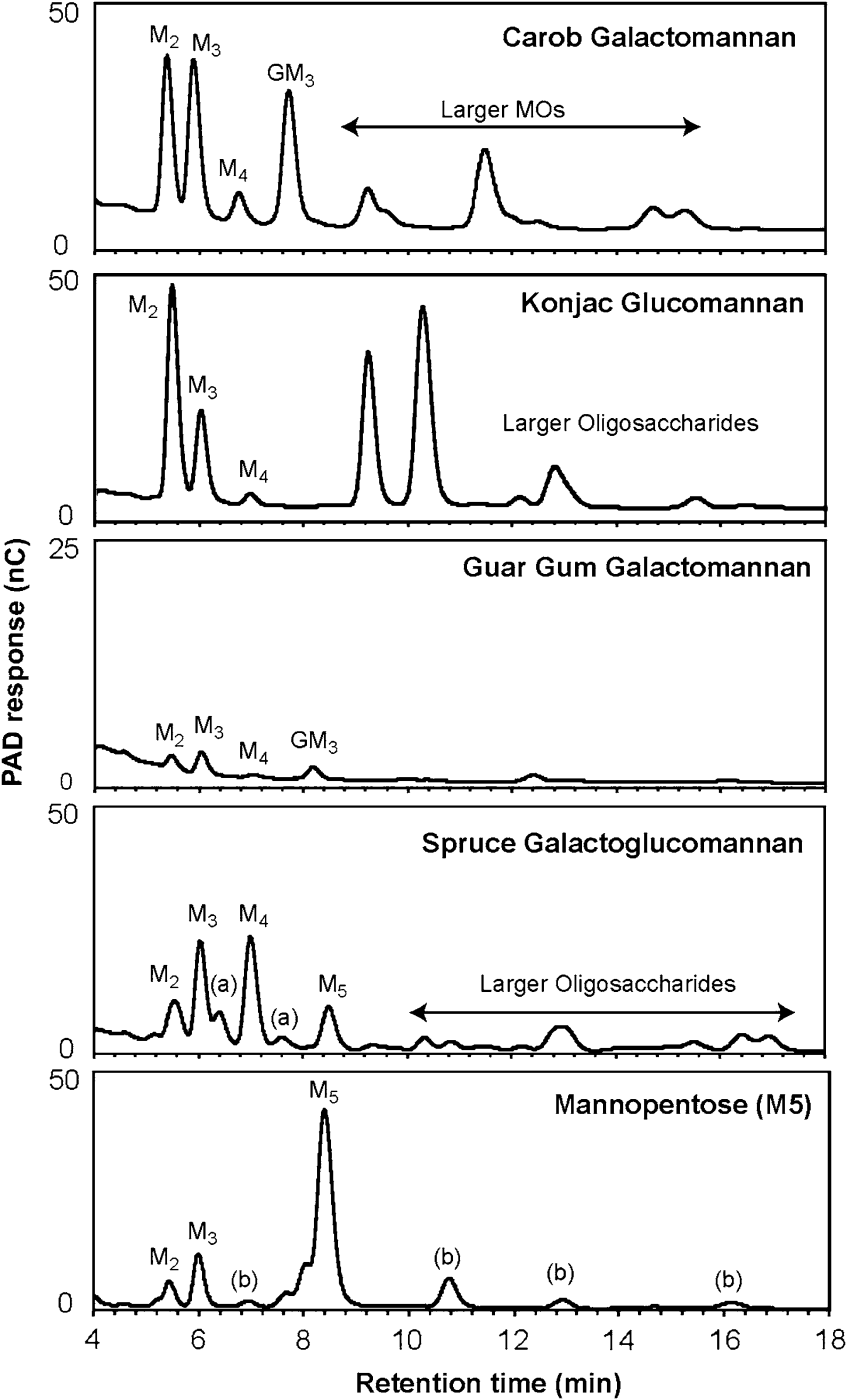
Oligosaccharide profiles using HPAEC-PAD after enzymatic hydrolysis of different mannan oligo- and polysaccharides. *a* Unassigned hydrolysis products, *b* mannan oligosaccharides present in the blank

**Fig. 8:**
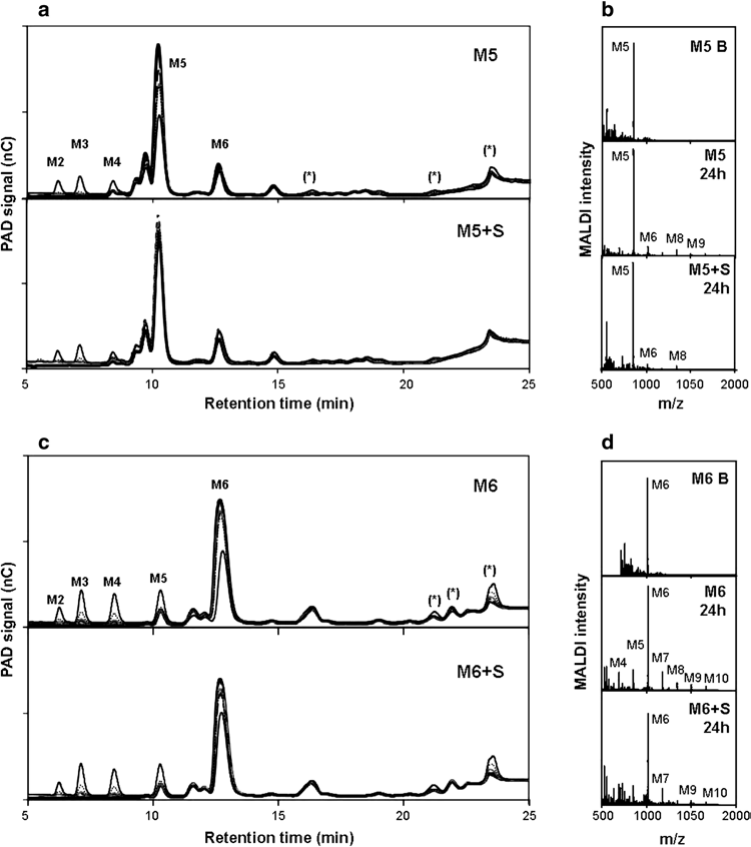
Evidence of transglycosylation activity by the AtMan5-1 enzyme. Incubation with (*inset* conditions): **a** HPAEC-PAD of mannopentaose (M5) and mannopentaose with increased salt content (M5 + S) different incubation times: () blank; () 15 min; () 30 min; () 1 h; () 4 h; () 24 h. Peaks with *asterisk* indicate transglycosylation products. **b** MALDI-ToF–MS analysis of mannopentaose blank (M5 B), mannopentaose after incubation for 24 h (M5 24 h) and mannopentaose with increased salt content after 24 h incubation (M5 + S 24 h). **c** HPAEC-PAD of mannohexaose (M6) and mannohexaose with increased salt content (M5 + S) different incubation times: () blank; () 15 min; () 30 min; () 1 h; () 4 h; () 24 h. Peaks with *asterisk* indicate transglycosylation products. **d** MALDI-ToF-MS analysis of mannohexaose blank (M6 B), mannohexaose after incubation for 24 h (M6 24 h) and mannohexaose with increased salt content after 24 h incubation (M6 + S 24 h)

To further investigate the AtMan5-1 mode of action, the AtMan5-1p hydrolysis products released from various mannan polysaccharides and mannan oligosaccharides were analyzed by HPAEC-PAD (Fig. 7; Table 3). Only the *Pichia*-produced recombinant protein was included in the study since the yield of soluble AtMan5-1e was relatively low. The major products from carob galactomannan depolymerization were M2, M3 and a galactomanno-oligosaccharide that can be assigned as a GM3. From konjac glucomannan hydrolysis the main identified oligosaccharide was M2, although M3 was also moderately abundant. Two abundant hydrolysis products could not be identified using HPAEC-PAD, although these fragments most probably consist of short glucomanno-oligosaccharides (Albrecht et al. 2011). Guar gum was not efficiently degraded by AtMan5-1p, although peaks for M2 and M3 could be identified. For the most complex mannan polysaccharide spruce galactoglucomannan, the hydrolysis yielded predominantly equal amounts of M3 and M4. Arabinoxylan, hydroxyethylcellulose (HEC) and xyloglucan incubated with AtMan5-1p yielded no hydrolytic products (data not shown). In addition to the polymer degradation product analysis, the hydrolysis of manno-oligosaccharides was investigated. Here, M3, M4 and G_2_M5 were not cleaved by AtMan5-1p (data not shown), whereas M2 and M3 were identified as the main products of M5 although not in high amounts. The digestion of M6 released M2, M3 and M4 among which M3 was the most abundant product. Comparison of the relative amounts of digested M5 and M6 revealed that the catalytic rate of AtMan5-1 towards M6 was 2.6 times higher than that of M5. The absence of products with a higher degree of polymerization (d.p.) than the examined oligosaccharides indicated that no transglycosylation reaction occurred. However, when in the substrate concentration of M5 was increased in order to favor transglycosylation the larger manno-oligosaccharides M6, M8 and M9 were observed indicating that recombinant AtMan5-1 is able to catalyze transglycosylation reactions in vitro at higher substrate concentrations (Fig. 8a, b). Moreover, transglycosylation at higher substrate concentrations occurs more favorably for mannohexaose (M6) than for mannopentaose (M5), suggesting a size dependence of the substrate on the transglycosylation capability (Fig. 8c, d). Salt addition had no apparent effect on the transglycosylation activity.

**Table 3:** Hydrolysis products released by AtMan5-1p from manno-oligosaccharides

Substrate (1 mM)	Products (mol/mol substrate)				
	M2	M3	M4	M5	M6
M5	0.0247	0.0324	0.0269	0.9161	0.0000
M6	0.0854	0.143	0.0905	0.0689^a^	0.7779

^a^This M5 content was already present in the M6 substrate as impurity and therefore does not constitute a hydrolysis product

## Discussion

Plant genomes contain a GH5_7 β-mannanase gene family comprising several members, which likely has expanded through gene duplication events (Cannon et al. 2004). The phylogenetic analysis of plant GH5_7 enzymes reveals two separate clades of moss mannanases suggesting that this subfamily in plants has evolved from two ancestral genes coding for GH5_7 enzymes. Members of the plant GH5_7 subfamily have probably evolved to hydrolyze various types of plant mannans, but may also perform transglycosylation (Schroder et al. 2006). Mannanases involved in the depolymerization of storage mannans must exhibit efficient hydrolytic activity, whereas those enzymes involved in modifying structural mannans presumably have a more delicate function. Although bioinformatic analyses such as the phylogenetic tree presented here provide clues to the evolution of plant GH5_7, it is essential to determine the catalytic properties of several plant mannanases to get a deeper understanding of the role of these diverse enzymes. The present study is the first, to our knowledge, characterization of a GH5_7 mannanase from the model plant *Arabidopsis thaliana*.

We tested heterologous expression of AtMan5-1 in one prokaryotic and one eukaryotic expression system, allowing us to conduct a comparative study of the biochemical properties of the recombinant enzyme produced in either *E. coli* (AtMan5-1e) or *P. pastoris* (AtMan5-1p). Effects of pH and temperature on enzyme activity were similar for both recombinant enzyme variants. The obtained pH optima close to 5.0 is in agreement with the reported pH optima for the characterized plant mannanases LeMAN4a and GmMAN1 (Lin et al. 2011; Schroder et al. 2006). The activity of both recombinant enzymes were unaffected by most of the screened metal ions. However, the activity increased in the presence of Co^2+^, and the enzymes were inhibited by the addition of Mn^2+^. Generally the influence of metal ions on mannanase activity varies with the organism, but interestingly, the soybean mannanase GmMAN1 has also been reported to be strongly inhibited by Mn^2+^ (Lin et al. 2011), and there are examples of bacterial mannanases whose activity was enhanced by Co^2+^ (Jiang et al. 2006; Li et al. 2008). The main difference noted between AtMan5-1e and AtMan5-1p concerned the thermal stability. The *E. coli*-produced enzyme was less stable at higher temperatures. SDS-PAGE analysis revealed glycosylation of the AtMan5-1p recombinant enzyme, and likely this modification contributed to the observed increased thermal stability of AtMan5-1p. The presence of predicted glycosylation sites in AtMan5-1 is in accordance with these results. It is known that removal of *N*-glycans from plant members of family GH16 reduces protein stability (Eklof and Brumer 2010). Besides influencing stability, glycosylation may also affect enzymatic activity. For instance, the importance of glycosylation for activity was recently reported for a poplar GH5_7 mannanase (Zhao et al. 2013). Nevertheless, in the present study only subtle activity differences were observed between AtMan5-1p and AtMan5-1e. It is not clear whether plant mannanases are glycosylated in general, but tomato mannanases involved in fruit development do not seem to be glycosylated (Schroder et al. 2006).

The substrate preferences varied slightly for the two recombinant forms of AtMan5-1. Both enzymes showed broad specificity towards structurally different mannans and efficiently degraded carob galactomannan, konjac glucomannan and spruce galactoglucomannan, demonstrating that AtMan5-1 is capable of hydrolyzing mannans with a backbone strictly consisting of mannoses as well as mannans having a backbone composed of both mannose and glucose units. Even though glucomannan was depolymerized, the data did not reveal whether AtMan5-1p cleaved only mannose–mannose, glucose–mannose or both glycosidic linkages. Previously, it has been shown that the native LeMan4 and HvMAN1 display similar mannanase activities on carob galactomannan and glucomannan (Carrington et al. 2002; Hrmova et al. 2006). In fact, the feature of degrading both glucomannans and less branched galactomannans (i.e., carob galactomannan) may be common to most GH5 mannanases. A noteworthy structural-based investigation revealed that GH5 mannanases have the capacity to bind both mannose and glucose in at least two subsites (Tailford et al. 2009). For guar gum only low level of hydrolysis by AtMan5-1 was detected. Apparently, a mannan-type with a high degree of galactose side-chains, such as guar gum (galactose:mannose ratio, 1:2), is not easily accessible to AtMan5-1. Thus, AtMan5-1 discriminates between mannans with different degrees of branching, and the still detectable hydrolysis is probably restricted to unsubstituted regions of the guar gum polymer. The determined catalytic efficiency (*k*_cat_ */K*_m_) of AtMan5-1e was slightly higher than for AtMan5-1p. Again, the apparent differences are probably a consequence of AtMan5-1p glycosylation. The observed catalytic efficiency for AtMan5-1 using carob galactomannan as substrate was approximately ten times higher compared to what was reported for GmMAN1 (Lin et al. 2011), but 10 times lower than the value measured for HvMAN1 (Hrmova et al. 2006). On the contrary, the (*k*_cat_ */K*_m_) was approximately 3–5 orders of magnitude lower compared to the catalytic efficiency numbers obtained for most fungal mannanases (Fu et al. 2010; Do et al. 2009; Couturier et al. 2011). The lower catalytic efficiency of AtMan5-1 compared to the numbers reported for fungal enzymes, probably reflects that AtMan5-1 belongs to the group of plant mannanases that are designed to modify and not completely degrade cell wall mannans. In contrast, the barley GH5_7 enzyme, which has a catalytic efficiency comparable with the fungal mannanases, is presumably involved in biological processes requiring potent depolymerization of mannan substrates.

The examination of AtMan5-1p hydrolysis products confirmed the results from the substrate specificity analysis and also demonstrated that the mannanase is a specific *endo*-acting mannanase. No enzymatic activity was detected when arabinoxylan, hydroxyethylcellulose (HEC) and xyloglucan were tested as substrates, but all mannan polysaccharides except guar gum were efficiently degraded with M2, and M3 as major hydrolysis products. The carob galactomannan hydrolysis product pattern obtained was similar to the hydrolytic cleavage profile of the same substrate incubated with a purified barley mannanase (Hrmova et al. 2006), including a released galactomanno-oligosaccharide with a d.p. of 4 identified as a galactosyl-branched mannotriose (GM3) in the barley study. The absence of products released from M3 and M4 incubated with the AtMan5-1p revealed the requirement of binding to at least five subsites for efficient hydrolysis to occur. Furthermore, the M6 oligosaccharide was hydrolyzed more efficiently compared to M5 which indicates that the substrate-binding site contains a minimum of six subsites. This is in line with the 5–6 subsites reported for other GH5 and GH26 β-mannanases (Anderson et al. 2008; Tailford et al. 2009). The major end products from M5 hydrolysis were M2 and M3, whereas the degradation pattern of M6 revealed M2, M3 and M4 as final products with M3 as the most abundant manno-oligosaccharide. To investigate if the substrate-binding site could accommodate a manno-oligosaccharide substituted with bulky galactosyl branches, a G_2_M5 oligo was incubated with AtMan5-1p. As expected no hydrolysis products were detected, indicating that the substrate-binding cleft was too narrow to fit galactose side chains in most subsites. Thus, AtMan5-1 can only cleave unsubstituted mannan domains. Binding to various mannan substrates by endo-β-mannanases are often hindered by galactose decorations and restricted to attack blocks of 3–5 unsubstituted mannopyranosyl residues in a row (Stalbrand et al. 1993; Katrolia et al. 2013). The observed manno-oligosaccharides with a d.p. larger than the examined oligosaccharide substrates suggested that transglycosylation events had occurred. However, transglycosylation products were only observed, when the substrate concentration was increased. AtMan5-1 possesses an arginine positioned at the +2 subsite, which has been proposed to play a significant role in giving rise to transglycosylation ability (Rosengren et al. 2012). AtMan5-1 is closely related to, and in the phylogenetic analysis located in the same clade as LeMAN4a, an enzyme with a demonstrated capability of transglycosylation (Schroder et al. 2006), and therefore such an activity was also expected for AtMan5-1. However, there are differences in the substrate-binding cleft between the two enzymes, especially around subsites +1 and −3, but apparently AtMan5-1 still is capable of acting as a transglycosylase. So what is the biological role of AtMan5-1? Mining of public Affymetrix microarray data using Genevestigator gives hints for the function of this particular enzyme (Hruz et al. 2008). The transcript is most abundant in root and seed tissues, but intriguingly, the gene expression of *AtMan5*-*1* is frequently induced by drought, cold and high salinity stresses, as well as by the hormone abscisic acid (ABA) (data not shown). None of the other Arabidopsis mannanase genes are as distinctly up-regulated by these factors as AtMan5-1. Probably, AtMan5-1 modifies cell wall mannans in response to elevated levels of ABA caused by drought and high salinity, but whether the mode of action in vivo includes hydrolysis and/or transglycosylation remains to be investigated.

The acquired detailed knowledge of the catalytic properties of the Arabidopsis mannanase, AtMan5-1 belonging to GH5 subfamily 7 presented in this study, is a first step towards understanding the enzymatic degradation and regulation of mannan-type polysaccharides in Arabidopsis. The results indicate that AtMan5-1 is adapted to hydrolyze linear stretches of mannans and glucomannans, can catalyze transglycosylation reactions at high substrate concentrations, and the importance of glycosylation for thermal stability was revealed by comparing recombinant production of AtMan5-1 in two different expression systems. Since only a minor portion of plant mannan-active enzymes have been biochemically characterized, the present findings are not only valuable for the Arabidopsis community, but crucial for further exploration of the plant mannanase portfolio.
